# Lung Cancer Mortality and Radon Concentration in a Chronically Exposed Neighborhood in Chihuahua, Mexico: A Geospatial Analysis

**DOI:** 10.1155/2014/935380

**Published:** 2014-08-06

**Authors:** Octavio R. Hinojosa de la Garza, Luz H. Sanín, María Elena Montero Cabrera, Korina Ivette Serrano Ramirez, Enrique Martínez Meyer, Manuel Reyes Cortés

**Affiliations:** ^1^Centro de Investigación en Materiales Avanzados S.C., Complejo Industrial Chihuahua, Avenida Miguel de Cervantes 120, 31109 Chihuahua, CHIH, Mexico; ^2^Facultad de Ingeniería, Universidad Autónoma de Chihuahua, Circuito Universitario Campus II, 31240 Chihuahua, CHIH, Mexico; ^3^Facultad de Enfermería y Nutriología, Universidad Autónoma de Chihuahua, Circuito Universitario Campus II, 31240 Chihuahua, CHIH, Mexico; ^4^Instituto de Biología, Universidad Autónoma de México, Ciudad Universitaria, A.P. 70-233, 4510 Mexico DF, Mexico

## Abstract

This study correlated lung cancer (LC) mortality with statistical data obtained from government public databases. In order to asses a relationship between LC deaths and radon accumulation in dwellings, indoor radon concentrations were measured with passive detectors randomly distributed in Chihuahua City. Kriging (K) and Inverse-Distance Weighting (IDW) spatial interpolations were carried out. Deaths were georeferenced and Moran's *I* correlation coefficients were calculated. The mean values (over *n* = 171) of the interpolation of radon concentrations of deceased's dwellings were 247.8 and 217.1 Bq/m^3^, for K and IDW, respectively. Through the Moran's *I* values obtained, correspondingly equal to 0.56 and 0.61, it was evident that LC mortality was directly associated with locations with high levels of radon, considering a stable population for more than 25 years, suggesting spatial clustering of LC deaths due to indoor radon concentrations.

## 1. Introduction

Lung Cancer (LC) has been characterized as a serious global public health issue with a high mortality rate. Based on the figures of the GLOBOCAN project, until 2008, LC had an incidence of 1,608,000 new cases each year (12.7% of all new cancer cases); moreover, this tumor was more common among men with 1,092,000 cases, (16.5% of the total) and 516,000 cases (8.5%) among women [1].

Smoking habits are the main cause for the development of LC, according to the World Health Organization [2]; smoking increases between 5 to 10 times the risk of LC. In developing countries 80% of LC related deaths are attributable to tobacco consumption [3–5].

There is a controversy about whether there is an association between smoking and the effects of radon. Nonetheless, radon inhalation is considered the second risk factor for the development of LC [4–10].

Radon is a radioactive element, colorless, odorless, of natural origin, occurring in rocks and soil. This element results of two successive radioactive decays: initially uranium ^238^U decays producing radium ^226^Ra and this turns into radon ^222^Rn. Radon continues to disintegrate producing three elements—polonium, lead, and bismuth—known as the* progeny of radon* [11]. The nuclear decay continues until it forms a stable and nonradioactive* progeny*. During this process, the release of alpha, beta, and gamma radiation takes place. If radon and its* progeny* are inhaled, alpha particles can easily reach the epithelia cells in the lungs causing alterations to their DNA [12, 13]. Continued exposure to radon increases and/or potentiates the risk of LC, which, according to the USA National Cancer Institute [14], is the only type of cancer associated with the inhalation of radon. According to WHO, LC exhibits a latency period around 25 years and it is worthy of mentioning that radon causes 15% of the cases of LC worldwide [3].

The city of Chihuahua is located near Mexico's main uranium ores. This situation, together with the igneous rock substrate of the region, makes uranium concentration in the ground an assumed source of radon emanation [15–17].

The National Survey of Addictions in 2011 (ENA-2011) [18] established that Chihuahua and Ciudad Juarez, both cities in Chihuahua state, have an active consumption of tobacco among people of 12–65 years of age of 24.4%, a higher consumption when compared with the national prevalence of 21.7%. As a reference the ENA-2008 [19] reported a 27.6% prevalence of tobacco consumption among people of 18–65 years of age in the state of Chihuahua and a 20.6% national prevalence. Historically, the LC mortality rates in Chihuahua City have been the double of those of Ciudad Juarez [17], which suggests the existence of another risk factor in addition to consumption of tobacco for LC deaths.

Atmospheric suspended particles, ranked by diameter, can deposit into the lungs; therefore inhaling them is recognized as a potential source of serious lung diseases. Decay products of radon are solid and are easily adsorbed by atmospheric particles. In particular, the climatic characteristics of Chihuahua contribute to the occurrence of suspended particles with diameters below 10 microns and coupled to that, during winter the accumulation of indoor gases is anticipated due to poorly ventilated homes.

In Mexico, geographical information systems (GIS) have been rarely implemented to monitor spatial assessments in cancer mortality. Nevertheless, recently it has been possible to obtain the database from Mexican Secretary of Health with detailed address information of death cases by different causes. Such information permits application of GIS [20–24]. This source of information revealed that in 2010 the LC mortality rate in Chihuahua City was 2.4× and 2× greater than the national and that of Ciudad Juarez, respectively. Note that this information is given for each 100,000 inhabitants per year from 2004 to 2010.

The aim of this work was to observe the evolution of LC mortality rate in the city of Chihuahua in comparison to the national mortality rate and thus relate geographically LC deaths with the occurrence of radon.

## 2. Materials and Methods

This research was conducted in Chihuahua City, in two phases. On the first, an epidemiological analysis of mortality data was conducted (2004–2010), to compare the frequency of occurrence of LC cases in the population throughout the country versus the Chihuahua state and the cities of Chihuahua and Ciudad Juarez. In the second phase, Chihuahua City was selected for a detailed inspection, based on the data from previous studies [17, 25] and the results of the first phase. Indoor radon concentrations were measured with passive detectors randomly distributed in Chihuahua City, as part of this phase. A Geographic Information System (ArcGis) was used to perform spatial geographic analysis of estimation about the distribution of deaths due to LC, searching for spatial clusters that may be associated with the inhalation of radon through the estimation of its air concentration indoors. In both phases, criteria used to verify information reliability are described in the “*criteria*” section.

### 2.1. Phase 1

#### 2.1.1. Mortality Data

Mortality data used in this research was generated by the Secretaria de Salud de Chihuahua (Chihuahua health institute), [26]. An ecological design was applied [27]. Data from certificates for LC deaths that occurred in Chihuahua City during the period 2004–2010 were examined; this data has been encoded and validated by qualified personnel according to the rules of the 10th International Classification of Diseases [2] of the World Health Organization.

#### 2.1.2. Criteria

Deaths classified as C349 were identified in the database. The consistency between the classifications was analyzed. In order to perform the analysis, once the suitable cases are selected, only the segment of population older than 30 years old was chosen and classified by age, sex, year of death, and geographic location.

Annual death rates were obtained per each 100,000 inhabitants for the period (2004–2010), and a comparison was made between the rates of the state of Chihuahua and the cities of Ciudad Juarez and Chihuahua with national results.

### 2.2. Phase 2

Chihuahua City was chosen to investigate possible correlations between radon exposure and death rates for LC cause as result of Phase 1.

To setup the spatial database, the exact location of the residence address as stated in the death certificate was located. For this purpose, the cartographic product “2009 Economic Census, DENUE March 2011” [28], the online digital urban map [29], and the ROJI maps guide of Chihuahua City [30] were used. All of them include the number of household and street name (spatially referenced). For a second validation, Google Maps technology was used: once the household was located, the street view was used to verify the number and street name with Google Earth. The neighborhoods limits in Chihuahua City were defined with this software. The study area has 224 km^2^, with a population of 572843 inhabitants [31].

#### 2.2.1. Criteria

The households that did not meet the exact location (neighborhood, address, or street number) were discarded from the spatial analysis.

### 2.3. Radon Concentration Assessment

Radon concentrations were measured in previously randomly selected houses, along the city of Chihuahua, from 2010 to 2012, during winter season only. Canisters were distributed among students of Universidad Autonoma de Chihuahua, Centro de Investigacion de Materiales Avanzados and the Instituto Tecnologico de Chihuahua, which are educational institutions. Students' dwellings are located in different sectors of the city with different socioeconomic levels.

As reference for data control, the protocol of the US Environmental Protection Agency (EPA) 520/5-87-005 [32] was used.

Detectors:

- passive detector ID and place where it was located,
- initial and final weight,
- date and time of beginning and end of exposure at dwelling,
- date and time of radioactivity measurement,
- measurement time.

Detector placement:

- lower level, master bedroom,
- placed at bed level,
- no heating,
- away from window and water sources.

In order to determine the indoor air radon concentrations with passive detectors, diffusion barrier charcoal canisters (DBCC) were used. The radon activity concentration in a single room was determined by using DBCC during 4 days, measuring its adsorbed radon activity and dividing the result by a calibration function reported in [33]. Radon activity was determined by measuring gamma spectra from a certified source, a blank canister, and the corresponding exposed DBCC.

Radon concentrations of the sampling were georeferenced, and contour maps for Chihuahua City were generated using interpolation techniques. The assigned values to LC death locations were calculated with a weighted average of the values available at the radon concentration measurement points. Two common techniques were applied: log-normal Kriging (K) interpolation (the weighted average of the measured values, having the minimum variance, is the value assigned to the LC location), according to the methodology described by Zhu et al. [34] and Buttafuoco et al. [35]; and inverse-distance weighting (IDW) interpolation, using the power factor 2 (weight decreases as the square of distance from the radon measurement points increases) [36].

The “outliers” or atypical values in radon concentrations were considered carefully using methods based on the standard deviation. Any measured value out of the range (smaller or greater than the resulting limits) was discarded [37].

These geographical statistical procedures generate an estimated surface area based on a scattered set of points with radon concentration values of the indoor air. Thus, values can be estimated in areas where no measurements are taken, and a map of radon concentration for Chihuahua City was generated. The corresponding interpolated in the map radon concentration values were added to the spatial database of LC deaths for each of the household addresses. To each of the georeferenced deaths, estimated radon concentrations to which the person was exposed were assigned.

To estimate those dwell residential times, the historical growing urban areas in Chihuahua City were spatially delimited for years 1920, 1921–1930, 1931–1969, 1970–1981, 1982–1993, and 1994–2012, using the geographic layers from the National Statistics and Geography Institute (INEGI) [38]. This information allowed the establishing of a reasonable time of LC latency for areas within the city and the verifying of the possibility of a cluster for the visually analyzed areas.

### 2.4. Data Analysis

ArcGis statistical package was used for spatial clustering search. To identify and measure auto-correlations in both models, the estimated values of radon concentration were introduced for each of the LC death addresses. The association between radon concentrations and LC death cases (through estimating the dispersion) was obtained by calculating the Moran's *I* index [39, 40] (see Appendix A). Moran's *I* index values vary between +1 and −1. The first value means a perfect positive correlation, the second one—a perfect negative correlation, and the zero represents a completely random spatial pattern. It was verified if these clusters met the criteria of the 25-year latency by visually reviewing the map of historical growth.

Finally, a risk exposure by radon inhalation classification was obtained and represented. In order to unify criteria, a consensus map was produced by averaging the value of interpolations IDW and Kriging. The geostatistical basic area units (AGEB) of INEGI were used to quantify the urban population by block. AGEB was optimal for zoning, looking for homogeneity in distribution, concentration, density, or centralization of the population in a metropolitan area [41].

According to radon concentrations, a risk exposure classification was suggested, considering four categories: low (0–74 Bq/m^3^), medium (74–222 Bq/m^3^) high (222–370 Bq/m^3^), and very high risk >370 Bq/m^3^) for visual representation of information, unifying EPA and working group criteria for this area [42, 43].

**Figure 1:**
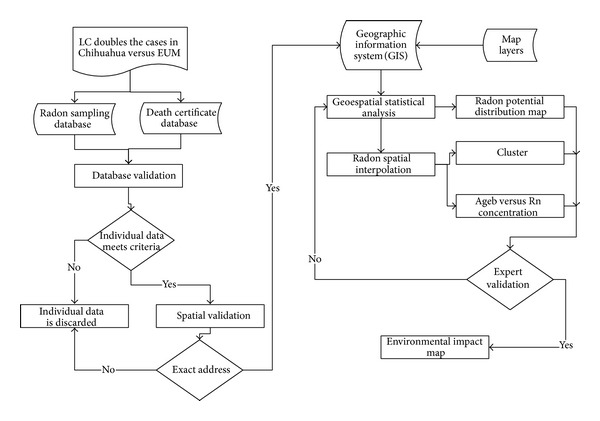
Working flowchart for the analysis of the databases. The criteria and methodology applied are described in the corresponding unit.

The process of integration of the entire methodology is described in a flowchart (Figure 1).

## 3. Results

During the period of 2004–2010 within the state of Chihuahua, LC cases were *n* = 2499, of which 1442 were men, 699 were women, and 358 showed no gender data. The mortality rate for the population of Chihuahua City increased in 1.3% (14.08% in 2004 and 14.27% in 2010), whereas in Ciudad Juarez it increased in 10% (6.7% in 2004 and 7.3% in 2010). In this context, the national LC mortality rate followed a low trend, decreasing in 9% (6.6% in 2004 and 6% in 2010; see Table 1).

The standardized mortality rate per LC 2010 in the city of Chihuahua was 2× greater than in Cd Juarez (which has twice the population of Chihuahua) and 2.4x greater than in México.

The largest number of LC deaths (*n* = 727), classified by age group, is in Chihuahua City. These defunctions are distributed as follows: 8% < 50 years, 50 years ≤ 22% ≤ 65 years, and 70% > 65 years (Table 2). Within these cases, 171 met all the criteria for spatial analysis (23%). The selection process for LC death addresses is presented in Appendix B.

**Figure 2:**
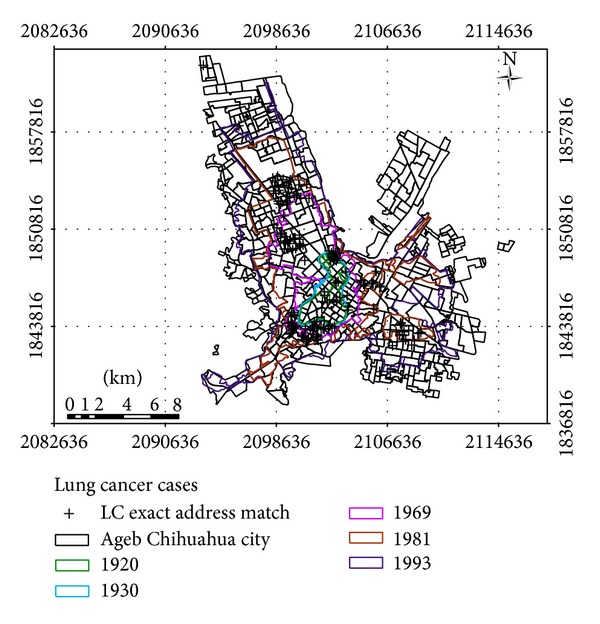
Historical growth of Chihuahua City versus LC deaths.

Historic city growth analysis revealed that 83% of LC deaths are in specific areas of city between the years 1969 and 1981 (see Table 3, Figure 2).

The average indoor radon concentration, sampled with canisters, in Chihuahua City was 249 Bq/m^3^, over *n* = 118 (Appendix C) with a minimal value of 0.3 to a maximum value of 1931.3 Bq/m^3^. Results of outliers' analysis of the 118 measurements indicated nine extreme values of radon concentration above 585 Bq/m^3^. These values were not taken into account to generate radon concentration interpolation maps through Kriging and IDW techniques [37]. Thus, there were only *n* = 109 values of radon activity concentrations taken as input for interpolations, with an average of radon concentration equal to 167.1 Bq/m^3^, with a minimal value of 0.3 to a maximum value of 585 Bq/m^3^.

**Figure 3:**
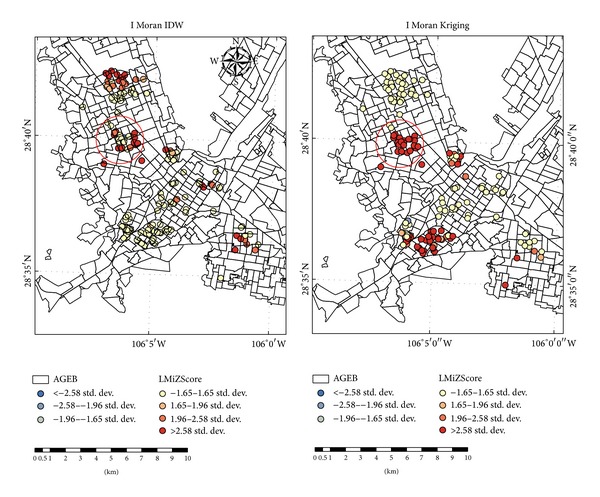
Cluster of LC deaths in Chihuahua City. LMiZScore means Local Moran Index Z score.

Moran's *I* index was obtained for the two types of interpolation results of the radon concentration in LC death addresses. Moran's *I* values were 0.61 (*P* < 0.01) for Kriging interpolation and 0.56 (*P* < 0.01) for IDW, respectively. The common area of the 2 maps was suggested as a cluster of LC deaths probably associated with high exposure to radon inhalation and it was delimited by a red circle (see Table 4 and Figure 3).

The number of LC cases, considering the four risk categories was: low (0 cases); medium *n* = 137 deaths, mean radon concentration = 169.5 ± 23 Bq/m^3^; high *n* = 33 deaths, mean radon concentration = 243.8 ± 21 Bq/m^3^; and very high risk *n* = 1 death, mean radon concentration = 445.9 Bq/m^3^.

The proposed cluster is characterized as follows: *n* = 28, with 22 men with a mean age of 70.9 ± 11.7 (34–91 years) and 6 women with a mean age 70.5 ± 8.9 (34–91 years). The radon activity concentration interpolated by Kriging was 250 ± 36 (178–392) Bq/m^3^ and IDW was 250 ± 63 (163–496) Bq/m^3^. These average values of radon activity concentrations correspond to the high exposure risk category.

**Figure 4:**
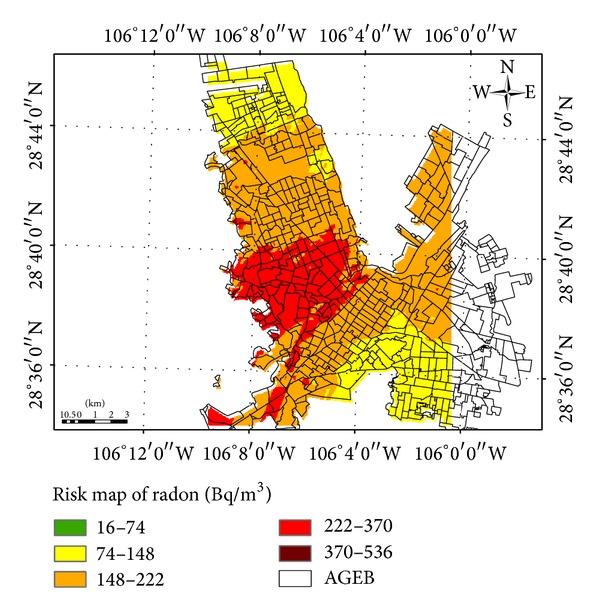
Radon exposure risk categories (Bq/m^3^) versus AGEB (total population).

The risk map of radon potential distribution was performed, assigning categories to the basic statistical units in Chihuahua City (AGEBs) [28] (see Table 5, Figure 4).

## 4. Discussion

In Chihuahua state LC death rates are higher than national rates and they show differences within the state territory. The rates show a great contrast when comparing among the populations of the two main cities (Ciudad Juarez and Chihuahua).

According to the national surveys of addictions from 2008 and 2011, in this ecological research we may conclude that no differences were found between the prevalence of tobacco consumption in the cities of Chihuahua and Ciudad Juarez. Tobacco is not considered a confounder but an independent predictor of LC. The number of LC deaths as the basic cause per age is the highest within the population over 65 years old in Chihuahua City. This is in agreement with other studies, which have encountered that the radon concentration in Chihuahua is higher than in Ciudad Juarez [17, 26].

The LC deaths in the GIS spatial database (*n* = 171) were mainly distributed into two historic areas of city growth. As a matter of fact, 42.1% of LC death cases were related to houses that were inhabited since 1969, whereas 40.9% of the cases were located in residences built and occupied in 1981.

Moran's *I* indexes obtained, well above zero for both interpolation methods, rule out the hypothesis of randomness, thus confirming the presence of a structural spatial dependence (depending on the distance). The cluster of LC deaths with significant high values (*P* < 0.01) is located in the North of the city and is represented with a red circle in the area of overlapping of the two maps generated (Moran's *I* versus Kriging or IDW). It is proposed as a cluster of LC deaths possibly associated with radon inhalation exposure. The estimated population living in this area is of 26,395 inhabitants: 12 425 men and 13 970 women.

It is the first time that this kind of spatial study is carried out in the city of Chihuahua, where high radon levels, as well as high rates of LC deaths, have been previously reported.

The risk map shows the potential distribution of radon. It is recommended as a tool for decision makers, which never must replace radon monitoring in dwellings through the use of active or passive detectors [43].

As a potential weakness to this research, the ecological fallacy has been considered [44]. The data used is valid within population levels, though they may vary at clinical level due to the habits of the individuals and genetic matters. One could argue that globally the association could be altered by the poor measurement to the response variable, which in this research was lung cancer. However, because this response is the result of an accurate histological diagnosis of high quality, it does not admit possible error. Therefore, it is considered that this association is not affected by performing an ecological measurement [45–47]. Other study limitation would be that the only information that exists about permanency of inhabitants in the cluster area is the one provided by the urban development authorities of the city of Chihuahua and the knowledge of the authorities of this article. This information allows us to affirm that the population was very stable up to 2010, when the public safety issues in the state caused address changes. Because this event is recent, these movements do not affect the data presented.

The measurement of the concentration of radon using canisters during winter allows the verifying of the houses where accumulation of radon was present. However, in order to calculate doses it would be necessary to perform measurements during the whole year. As a result this study is able to reject the null hypothesis of the random grouping of LC and that there is a probability that radon inhalation is a cause.

It would be worth analyzing smoking as independent predictor to the exposure to radon. It is suggested to continue in this line of research to get more accurate measurements.

There are studies that have examined the relationship between cancer and radon in soil, groundwater, and air in Mexico, both indoors and outdoors, as part of geophysical studies and to estimate effective doses as a result of radon exposure but there are not any other documented clusters. This detection of radon has mainly been performed with solid-state nuclear track detectors (SSNTD) and with active detection devices based on silicon detectors or ionization chambers [48, 49].

## 5. Conclusions

The average value of indoor radon concentration in Chihuahua City was 249 Bq/m^3^, with concentrations varying among 0.3–1931.3 Bq/m^3^. Previous studies of indoors radon levels indicated an average of 136 (Bq/m^3^) [17], and an evident increase was observed. After verifying that the LC death rate of Chihuahua City is 2× greater than the national average, a different methodology to analyze historical information of LC deaths was proposed. The geospatial association between LC and indoor radon concentrations in the proposed cluster has been statistically proven; and the technical foundation to prevent population exposure in different regions has been pointed out by using risk maps. Therefore, it is proposed to include radon mitigation actions in the local regulation of construction, coupled to compulsory monitoring and remediation in existing buildings throughout the city.

We recommend a mass media awareness campaign regarding the source and effects of radon and simple measurements to prevent its exposure and continuing with the current antismoking campaigns.

## Figures and Tables

**Figure 5:**
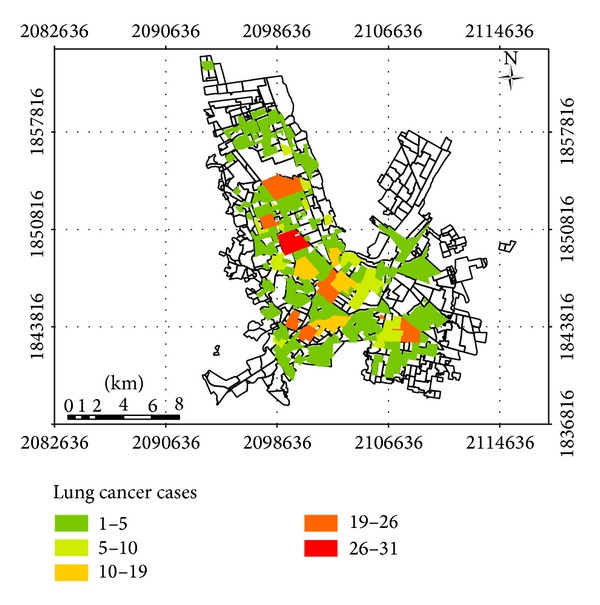
Chihuahua City map with the location of deaths by LC between 2004 and 2010. Lung cancer cases mean the number of deaths by neighborhood, according to the information provided by Secretary of Health. Colors allow appreciating the neighborhoods with more deaths by LC.

**Figure 6:**
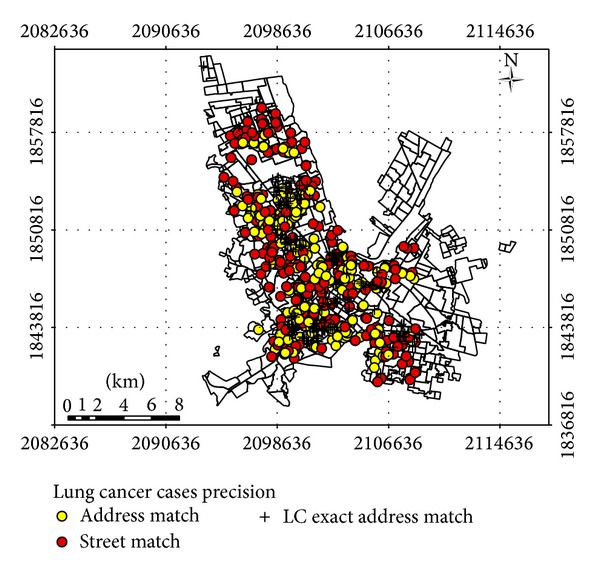
LC death's address verification in Chihuahua City. The verified addresses are shown by the “+” symbol (*n* = 171); the home address that has a problem with some information is indicated in yellow and red circles (*n* = 139 and *n* = 368).

**Table 1:** LC mortality rates of Chihuahua City, Ciudad Juarez, and México.

Year	Chihuahua	Juarez	México
2004	14.1	6.7	6.6
2005	11.3	8	6.8
2006	14.8	7.1	6.5
2007	13.6	6.8	6.3
2008	13.1	6.1	6.3
2009	11.3	5.8	6.2
2010	14.3	7.3	6

Source: Secretary of Health/Directorate General of Health Information in Chihuahua. Estimates are based on Population Projections for México 2005–2030, CONAPO, II Population and Housing Census 2005, and the Population and Census 2010. LC: lung cancer. The mortality rate is given per 100 000 persons.

**Table 2:** Deaths by lung cancer as the underlying cause in Chihuahua City in population older than 25 years.

Age	2004		2005		2006		2007		2008		2009		2010		Total
	M	F	M	F	M	F	M	F	M	F	M	F	M	F	
25–29	1	0	0	0	0	0	0	0	0	0	0	0	0	1	2
30–34	0	0	2	0	0	1	1	1	0	0	0	0	0	0	5
35–39	1	0	0	0	1	0	0	0	1	1	1	0	0	1	6
40–44	3	1	2	1	4	0	2	0	3	0	3	1	4	2	26
45–49	2	1	1	1	2	1	1	1	0	1	1	2	1	3	18
50–54	1	2	1	1	6	2	5	0	3	3	1	2	3	0	30
55–59	5	1	3	3	6	5	8	4	7	0	3	2	4	2	53
60–64	11	3	9	1	14	0	8	3	6	2	6	5	7	4	79
65–69	9	5	9	7	16	9	10	2	13	7	5	3	13	5	113
70–74	13	8	13	6	5	4	13	8	21	6	10	6	10	5	128
75–79	16	10	10	4	16	4	12	5	10	4	13	6	19	5	134
80–84	7	2	3	4	4	5	10	8	7	6	11	1	8	6	82
85–89	1	1	1	2	3	2	3	1	3	0	4	2	5	3	31
90–94	1	1	0	2	2	2	0	0	0	0	3	0	2	3	16
95–99	0	0	0	0	1	0	0	1	0	0	1	0	1	0	4

Total	71	35	54	32	80	35	73	34	74	30	62	30	77	40	
	106		86		115		107		104		92		117		727

Source: Secretary of Health/General Directorate of Health Information in Chihuahua. Estimates are based on Population Projections for México 2005–2030, CONAPO, II Population and Housing Census 2005, and the Population and Census 2010. Lung Cancer is classified by code C349. M: male; F: female.

**Table 3:** Historical growth of Chihuahua City versus Bq/m^3^ estimates for deaths georeferenced.

Year	*N*	Bq/m^3^	Age of death (mean)	Age of death (min–max)
1920*	17	245 ± 29	66 ± 16	30–85
1930	4	256 ± 43	70 ± 21	39–84
1969	72	333 ± 90	69 ± 13	28–91
1981	70	257 ± 57	68 ± 12	34–92
1993	8	236 ± 14	64 ± 15	39–84

*Downtown.

**Table 4:** Spatial correlations.

Interpolation	Spatial autocorrelation *I* Moran
Kriging 109	*I* = 0.61, *Z* = 20.9, *P* = 0.01
IDW 109	*I* = 0.56, *Z* = 19.85, *P* = 0.01

**Table 5:** Radon exposure risk categories (Bq/m^3^) versus AGEB (total population).

Description	Men	Women	Total
Low risk (4–74 Bq/m^3^)	3 443	3 348	6 791
Medium risk (74–148 Bq/m^3^)	30 358	31 938	62 296
High risk (148–370 Bq/m^3^)	214 089	224 398	438 487
Very high risk (>370 Bq/m^3^)	67 210	73 537	140 747

Notice: the consensus map was used as a basis to quantify the population.

**Table 6:** Radon concentration values (RC), in Bq m^−3^, obtained by measurements in random selected dwellings at Chihuahua City, Chihuahua, Mexico. *X* and *Y* are geographic longitude and latitude, respectively.

RC	*X*	*Y*
150	−106.143	28.689255
203.5	−106.121	28.738078
264.1	−106.134	28.683602
87.99	−106.143	28.692722
159.5	−106.15	28.692506
134.9	−106.15	28.655593
236.4	−106.12	28.677433
504.1	−106.1	28.595789
274.2	−106.11	28.726177
132.5	−106.13	28.748513
185.4	−106.1	28.719461
128.5	−106.13	28.7407
693.1	−106.09	28.634252
299.6	−106.02	28.649546
205.8	−106.01	28.657465
1778	−106.13	28.67081
142.5	−106.11	28.61914
699	−106.11	28.61914
585	−106.09	28.656286
438.9	−106.14	28.683809
197.7	−106.08	28.663992
80.2	−106.14	28.712529
191.4	−106.14	28.682457
262.2	−106.13	28.701008
153.2	−106.13	28.698289
216.5	−106.15	28.721249
194.6	−106.09	28.632977
78.05	−106.15	28.703597
403	−106.14	28.717492
363.9	−106.13	28.625798
68.74	−106.17	28.772309
295.6	−106.13	28.625798
99.7	−106.04	28.62749
41.03	−106.12	28.73795
229.3	−106.14	28.714763
111.6	−106.15	28.689209
21.79	−106.15	28.742
33.21	−106.15	28.71678
94.14	−106.11	28.61702
161.1	−106.08	28.651598
91.36	−106.092956	28.638554
80.95	−106.146391	28.64963
151.67	−106.040566	28.599516
314.12	−106.067193	28.643522
133.81	−106.091596	28.619658
185.97	−106.016807	28.652503
197.28	−106.118718	28.722543
381.53	−106.146998	28.703687
55.62	−106.044523	28.636603
89.95	−106.154496	28.593884
65.89	−106.010599	28.670822
186.93	−106.113151	28.684938
204.38	−106.067193	28.643522
147.85	−106.067167	28.64439
408.1	−106.103371	28.682912
28.39	−106.103371	28.682912
8.07	−106.103371	28.682912
14.19	−106.103371	28.682912
187.71	−106.103371	28.682912
44.71	−106.103371	28.682912
63.47	−106.103371	28.682912
4.65	−106.103371	28.682912
67.8	−106.103371	28.682912
52.01	−106.103371	28.682912
30.54	−106.103371	28.682912
40.98	−106.103371	28.682912
15.85	−106.103371	28.682912
195.77	−106.090843	28.717466
143.45	−106.076983	28.649922
515.22	−106.09335	28.660756
524.49	−106.1174	28.651103
69.32	−106.067833	28.632944
130.8	−106.086342	28.61615
28.39	−106.094665	28.595761
8.07	−106.08248	28.623518
14.19	−106.1315	28.687614
187.71	−106.113949	28.707465
4.65	−106.129525	28.695041
67.8	−106.150203	28.736901
52.01	−106.09725	28.619133
40.98	−106.149393	28.709121
15.85	−106.144804	28.720217
1931.28	−106.09963	28.623148
1782.98	−106.142494	28.68847
622.13	−106.133314	28.693821
149.96	−106.142903	28.689255
374.72	−106.088075	28.663658
93.64	−106.103453	28.666872
101.85	−106.098758	28.659889
251.35	−106.107078	28.656819
328.56	−106.112906	28.669583
394.27	−106.105831	28.672308
708.65	−106.072947	28.666097
195.77	−106.090843	28.717466
143.45	−106.076983	28.649922
515.22	−106.09335	28.660756
423.31	−106.088845	28.635334
524.49	−106.1174	28.651103
69.32	−106.067833	28.632958
130.8	−106.086342	28.61615
232.73	−106.077064	28.709394
137.62	−106.010747	28.574078
1862.73	−106.145321	28.72253
328.93	−106.098631	28.623
278.98	−106.105603	28.614576
180.56	−106.010747	28.574078
1147	−106.032271	28.602279
130.8	−106.111032	28.675248
129.38	−106.014223	28.66454
120.34	−106.064356	28.663182
117.62	−106.091731	28.607276
51.59	−106.067816	28.674201
50.12	−106.067542	28.674784
26.33	−106.098946	28.718358
26.33	−106.058595	28.60697
17.39	−106.034878	28.602769
5.58	−106.095185	28.719983
0.37	−106.108291	28.663596

## Appendices

### A. Moran's Index

If a database contains a spatial representation of the information, the structure of this data is a model of spatial information [50]. The spatial autocorrelation (SA) is the mathematic demonstration of the concentration or dispersion of the values of a variable in a map. A spatial autocorrelation can be defined as “property of a set of data located on a geographical map showing a pattern of organization” [51]. The SA reflects the degree to which the value of a random variable *z*_*i*_ in a geographical unit is similar to the value of another random value *z*_*i*_ in neighboring geographic units. The present study analyzes the geographic distribution of a random variable radon concentration in homes and the variable deaths for LC localization.

#### A.1. Moran's Index

*I* Moran is a global statistic that shows the existence of local spatial agglomerations around greater or lower values of the mean of all observations, that is to say, verifies if the variables are grouped, scattered or have a random distribution [52–54]. This *I* Moran index verifies the null hypothesis, that is, that the radon data analyzed is a random sample obtained from one of the *n*! possible spatial distributions among the *n* locations of deceased's homes or vice versa. The definitions are as follows [55]:

(A.1) I=nS0∑i=1n∑j=1nwi,jzizj∑i=1nzi2S0=∑i=1n∑j=1nwi,jzi=I−E[I]V[I],where  V[I]=E[I]2−E[I]2, E[I]=−1n−1,

where *z*_*i*_ is the deviation of the *i*-value of variable from its mean value (*x*_*i*_ − 〈*X*〉), *w*_*ij*_ is the spatial weight between variable *i* and *j*, *n* is equal to the total number of variables, and *S*_0_ is the sum of all spatial weights.

If the values in the data set tend to group spatially, namely, the high values of the variables studied are close spatially and the lower values are close to other low values (grouped values), the Moran index will be positive. When the high values “reject” territorially other high values and tend to be surrounded by low values, the index will be negative (scattered values). If the positive values of the “cross products” balance the negative values of “cross products”, the index will be close to zero. The numerator is normalized by the variance in a way that the values oscillate between −1.0 and +1.0. Therefore, the proximity of the index to the value zero means randomness in the spatial relation.

The calculation is made with specialized software that also provides values of *P* that indicate if the differences with the randomness are statistically significant.

### B. Georeferencing Techniques Employed for Creating Lung Cancer Deaths Database

Due to difficult access to individual blocks or lots, 49 addresses were eliminated from Chihuahua's lung cancer decease database.

Localization and georeference techniques were utilized on the 678 remaining addresses: Google Earth's Apis https://developers.google.com/maps/?hl=es, Global Mapper y Arcgis. As a result it was possible to completely georeference 310 addresses; for the remaining 368 only the street address without the number was found (Figure 5).

However, when validating the 310 addresses with the 3 complete data (address number, street name, and neighborhood name) errors were found in the above mentioned databases. As a result we proceeded to validate one by one the existence of the address, looking the exact location with the minimum error possible.

With this manual validation, errors ranging from 5 to 822 meters were corrected for each of the addresses generated from the databases. Finally addresses where the exact number was not found were discarded. This procedure produced 171 addresses with an exact address match.

Figure 6 shows the addresses of deaths with verified data (*n* = 678) in the geostatistic units (AGEB). It was noticed that the most confirmed information was the “neighborhood” or “AGEB” and that only 171 deaths have verifiable information.

### C. Indoor Radon Concentrations in Chihuahua City

See Table 6.
